# Minimal Evidence of Inflammaging in Naturalistic Chimpanzee Populations

**DOI:** 10.1002/ajpa.70211

**Published:** 2026-02-09

**Authors:** Megan F. Cole, Melissa Emery Thompson, Nicole Thompson González, Eleanor Paskus, Joshua Rukundo, Rebeca Atencia, Alexandra G. Rosati

**Affiliations:** ^1^ Department of Anthropology University of New Mexico Albuquerque New Mexico USA; ^2^ Department of Anthropology Emory University Atlanta Georgia USA; ^3^ Department of Psychology University of Michigan Ann Arbor Michigan USA; ^4^ Integrative Anthropological Sciences University of California Santa Barbara California USA; ^5^ Chimpanzee Sanctuary and Wildlife Conservation Trust Entebbe Uganda; ^6^ Jane Goodall Institute Brazzaville Republic of the Congo; ^7^ Department of Anthropology University of Michigan Ann Arbor Michigan USA

**Keywords:** aging, health, human evolution, lifestyle, primates

## Abstract

**Objectives:**

Whereas chronic inflammation is a hallmark of aging in many human populations, inflammaging is reduced in groups characterized by frequent physical activity and diets low in processed foods. Since most biomarkers of inflammation require blood sampling, comparative data from our closest primate relatives have been derived from sedentary, captive primate populations whose processed diets are uncharacteristic of the wild.

**Materials and Methods:**

We evaluated aging profiles of inflammation and oxidative stress biomarkers derived from urine and serum samples in semi‐free ranging chimpanzees ( *Pan troglodytes* ) living in two African sanctuaries (*N* = 156 health checks, 73 individuals, ages 11–39 years), where diet and physical activity more closely approximates wild conditions than captive laboratory settings. We compared these to urinary markers from wild chimpanzees from Kanyawara, Kibale National Park, Uganda (*N* = 1849 time points, 50 individuals, ages 10–57 years), as well as published serum data from biomedical laboratories.

**Results:**

Serum inflammatory biomarker (CRP and IL6) levels in sanctuary chimpanzees were 2–10 times lower on average than those of laboratory chimpanzees. Compared to wild populations, acute immune activity (neopterin) and lipid peroxidation (isoprostanes) were higher in sanctuaries, while chronic systemic inflammation (suPAR) and DNA damage (OHdG) did not differ. We detected a significant but modest age‐related increase in one biomarker (suPAR) in the wild sample.

**Discussion:**

These results parallel recent findings from humans in demonstrating that chronic inflammation is not a natural consequence of aging but may rather be driven by environmental contexts that are mismatched to the evolutionary history of a given species.

## 1 Introduction

‘Inflammaging’—the accumulation of inflammation and oxidative stress over time—is a significant risk factor for morbidity and mortality of older adults, and as such, is considered central to the aging process (Ferrucci and Fabbri 2018; López‐Otín et al. 2023; Pizzino et al. 2017). Inflammation is particularly implicated in the pathogenesis of ischemic heart disease, the leading cause of death in the US, and covaries with related risk factors such as obesity and elevated proatherogenic blood lipid levels (Buckley et al. 2009; Kochanek 2024; Libby et al. 2002). However, healthy lifestyle factors such as regular exercise and low‐fat, plant‐rich diets can reduce oxidative damage and inflammation across the lifespan (Dai et al. 2008; Fisher‐Wellman and Bloomer 2009; Wawrzyniak‐Gramacka et al. 2021), to the extent that some small‐scale subsistence populations that rely heavily on foraging exhibit a remarkable resistance to heart disease, even into old age (Kaplan et al. 2017; Pontzer et al. 2018). Thus, it is widely hypothesized that cardiovascular diseases and other chronic degenerative diseases linked to inflammation are the consequences of an evolutionary mismatch between features of modern human environments and those that shaped the evolution of the human body (Eaton et al. 1988; Gurven and Lieberman 2020). If so, inflammaging itself may be a relatively novel phenotype rather than an inevitable consequence of aging. Given the stark variation across contemporary human populations, comparative data from closely related primate species are valuable to evaluate whether inflammaging is a novel or evolutionarily ancient aging phenomenon, but those comparisons should include contexts that approximate evolutionarily appropriate environments for those species. This presents a challenge since inflammation is most commonly assessed using serum biomarkers that are not readily obtained from wild primates. Here, we evaluate non‐invasive urinary biomarkers from wild chimpanzees ( *Pan troglodytes* ) and both serum and urinary biomarkers from semi‐free‐ranging chimpanzees to evaluate whether multiple biomarkers of inflammation and related markers like oxidative stress increase with age in naturalistic settings.

There are several physiological processes that are relevant to understanding inflammaging. First, the innate immune response is a cascading physiological pathway initiated by injury or infection, and inflammation is an essential element of this response. Briefly, neutrophils are first responders that ingest and destroy foreign bodies while simultaneously recruiting other immune cells like macrophages to the site (Janeway et al. 2001). Macrophages continue fighting infection and produce chemical mediators such as cytokines, including interleukins such as IL6, that respond to infections in part by stimulating inflammatory processes (Ahmed 2011; Medzhitov 2008). In response to these proinflammatory cytokines, C‐reactive protein (CRP) is also produced and amplified in large amounts (up to 1000‐fold) by the liver to remove pathogens (Sproston and Ashworth 2018). Elevated cytokines and spikes in CRP levels are therefore useful clinical markers for monitoring acute infection and inflammation. Inflammation is also intricately linked to oxidative stress, an imbalanced state of reactive oxygen species (ROS) relative to antioxidants (Maldonado et al. 2023). ROS are an endogenous byproduct of oxygen metabolism but are also generated in response to immune activation and inflammation. Moderate levels of ROS are beneficial for immune and cellular activity, but accumulation leads to damage of lipids, DNA, cells, and tissues, several byproducts of which can be measured in blood or urine (Pizzino et al. 2017). Oxidative damage also feeds forward on the inflammatory process by promoting activation of redox‐sensitive transcription factors, which in turn upregulates inflammatory signaling pathways (Chatterjee 2016).

Importantly, persistent activation of the immune system can result in chronic, low‐grade inflammation, and this pattern is often exacerbated during aging. People in post‐industrial societies regularly sustain clinically elevated CRP levels (> 3 mg/L (Pearson et al. 2003)) indicative of chronic, low‐grade inflammation that put them at risk for related morbidities. For example, in the Multi‐Ethnic Study of Atherosclerosis, CRP levels were 3.78 mg/L on average, even after excluding patients with cardiovascular disease (Jenny et al. 2010). A prominent predictor of chronic inflammation is obesity, since adipocytes elicit cellular stress and subsequently activate systemic inflammation (Coppack 2001; Day 2006; Lee et al. 2013). Chronic inflammation promotes obesity in a feed‐forward loop involving insulin resistance and metabolic dysfunction (De Souza et al. 2005; Gregor and Hotamisligil 2011). Systemic inflammation also plays a role in the pathogenesis of atherosclerosis and high blood pressure via narrowing of the blood vessels (which in turn causes a buildup of low‐density lipoproteins, or LDLs, and promotes further inflammation) (Libby et al. 2002). In the Global North, biomarkers of inflammation often show marked age‐related increases (Ferrucci and Fabbri 2018; López‐Otín et al. 2023; Pizzino et al. 2017). As such, chronic inflammation is a prominent risk factor for a number of chronic ‘diseases of aging’ such as cardiovascular disease, diabetes, and some cancers (Buckley et al. 2009; Ferrucci and Fabbri 2018). On the other hand, caloric restriction (reduced energy intake without malnutrition) improves health and survival in captive mammals including primates, and appears to have similar effects on the human healthspan (Mattison et al. 2017; Most et al. 2017; Wood and Sullivan 2022). Oxidative stress also generally increases during aging, as cellular repair function declines (Maldonado et al. 2023). ROS are further involved in the pathogenesis of cardiovascular disease and other age‐related diseases including cancer and neurological diseases (Esterbauer et al. 1993; Liguori et al. 2018; Pizzino et al. 2017). The interacting pathways promoting an accumulation of inflammation and oxidative stress with age are characteristic of the ‘inflammaging’ phenotype which is so common in industrialized populations that it has been defined as one of the ‘hallmarks of aging’ in humans (López‐Otín et al. 2023).

What are the causes of this inflammaging pattern? One hypothesis is that it reflects an evolutionary mismatch between the conditions in which our physiology evolved and modern, post‐industrial environments (Eaton et al. 1988; Gurven and Lieberman 2020). In this view, although it was biologically adaptive to store excess energy as visceral fat in our species' evolutionary past, the high‐calorie diets and frequent sedentism of many modern environments often promote a maladaptive inflammatory response. In line with this view, inflammation and oxidative status are influenced by lifestyle factors like diet and exercise, like many indices of health. Foods with high glycemic index and glycemic load values, such as saturated fats and carbohydrates, are consistently associated with high inflammatory biomarker levels, while plant‐based diets heavy in fruits and leafy greens, nuts and seeds, and lean protein (e.g., Mediterranean diet) are generally anti‐inflammatory (Galland 2010; Lee et al. 2013). Mediterranean and similar diets are likewise high in dietary antioxidants and flavonoids, which protect against oxidative stress (Dai et al. 2008). While physical activity induces acute oxidative stress, regular exercise also leads to long‐term up‐regulation of antioxidant defenses (Fisher‐Wellman and Bloomer 2009) as well as protection against systemic inflammation via reduction of insulin resistance and adipose tissue (Beavers et al. 2010; Petersen and Pedersen 2005). These interventions even prove effective in attenuating the process of inflammaging (Wawrzyniak‐Gramacka et al. 2021).

Small‐scale subsistence societies are a compelling illustration of these lifestyle effects, as they have low prevalence of chronic cardiovascular disease and associated risk factors, even into old age (Kaplan et al. 2017; Pontzer et al. 2018; Raichlen et al. 2017). This has been attributed to high levels of moderate to vigorous physical activity and diets low in processed foods, low in saturated fats, and high in fiber. Accordingly, low CRP (below the clinically ‘elevated’ threshold of 3 mg/L) has been reported on average for the Shuar (forager‐horticulturalists of Ecuador, 0.5 mg/L (McDade et al. 2012)), rural Ghanaians (agriculturalists, 0.8 mg/L (Eriksson et al. 2013)), the Hadza (hunter‐gatherers of Tanzania, 1.5 mg/L (Raichlen et al. 2017)), and the Kitava (horticulturalists of Papua New Guinea, 0.47 mg/L (Carrera‐Bastos et al. 2020)). Such groups generally show minimal to no age‐related increase in CRP (Eriksson et al. 2013; Kaplan et al. 2017; McDade et al. 2012). However, some populations with high immune burden can show elevated inflammation. For example, the Tsimane (forager‐horticulturalists of the Bolivian Amazon, 3.7 mg/L (Kaplan et al. 2017)) experience repeated helminth infection across the life course and show elevated interleukins, white blood cell counts, and immunoglobulins (Blackwell et al. 2016; Kaplan et al. 2017). In line with this, a recent study reported that the Tsimane also show a modest but significant age‐related increase in IL6, but not in other pro‐inflammatory markers such as IL1ß and TNFα; meanwhile, inflammaging was accelerated in the neighboring group of Moseten who are characterized by increased market integration and associated health risks (Aronoff et al. 2025). Yet even in the face of chronic inflammatory activation, the Tsimane maintain exceptional cardiovascular health, suggesting that helminths may be cardioprotective, or that lifestyle benefits outweigh the negative effects of inflammation (Gurven et al. 2009, 2016).

**FIGURE 1:**
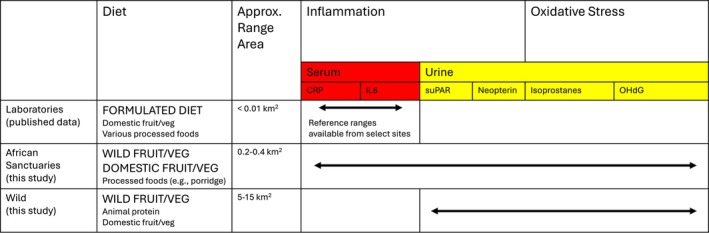
Diet, ranging area, and biomarkers available from laboratory, sanctuary, and wild chimpanzees. Dietary items in large, bold print are the majority items in the diet, with minor components in small print. As of 2010, laboratory guidelines advise that dark, leafy greens make up 50% of chimpanzee diets to supplement commercial primate ‘biscuits’ (Ross and McNary 2010). However, most reports available for comparison predate this change and represent a time when biscuits fed *ad libitum* comprised the vast majority of diet (Clay et al. 2022; King 1978; Knapka 2003). Domestic fruit/vegetables are not provisioned to wild chimpanzee diets but come from occasional feeding (< 5%) on farms bordering the forest.

The mismatch hypothesis would predict that (a) other long‐lived primate species, who share an extended evolutionary history with humans and many genetic and physiological similarities, would not exhibit inflammaging phenotypes in their natural environments; and (b) lifestyle conditions that parallel the effects of industrialization on human populations, namely sedentism, a high proportion of calorific processed foods, and increased body fat, would increase levels of inflammation during aging. Variation in chimpanzee living environments makes them an ideal study system through which to test this idea (see Figure 1 for diet and ranging summary). Wild chimpanzees are ripe fruit specialists who fall back on plant pith when fruit is scarce (Conklin‐Brittain et al. 1998; Wrangham et al. 1998). In home ranges spanning ~5–15 km^2^ (Basabose 2005; Green et al. 2020; Mitani et al. 2010; Newton‐Fisher 2003; Rutherford 2021; Vieira et al. 2019), they travel 2.1–4.2 km/day on average and climb an additional 113 vertical m/day (Herbinger et al. 2001; Pontzer and Wrangham 2006; Wrangham 1977). In captivity, in contrast, chimpanzee diet and activity patterns range substantially based on local facility and welfare guidelines. Historically, U.S. laboratory chimpanzees have primarily been fed commercial primate chow *ad libitum* (Clay et al. 2022; King 1978) and have been housed in small enclosures in biomedical research facilities: the National Institutes of Health recommends a minimum of 250 ft^2^ per individual (National Institutes of Health 2014). Chimpanzees living in African sanctuaries, on the other hand, more closely approximate their wild counterparts in terms of lifestyle, physiology, and behavior (Rosati, Emery Thompson, et al. 2023; Rosati et al. 2013; Rosati, Sabbi, et al. 2023; Wobber and Hare 2011). They range in large, forested enclosures (15–40 ha) where they can forage on wild foods, and their diets are supplemented with provisioned domesticated fruits and vegetables but do not contain the highly processed foods that are common in laboratory contexts (Pan African Sanctuary Alliance 2009; Stokes et al. 2018).

Accumulating evidence shows that the kinds of lifestyle effects on cardiovascular health and aging that are common in humans are also shared with other great apes. For example, chimpanzees living in African sanctuaries have lower body weights, lower levels of proatherogenic blood lipids such as cholesterol, and lower blood pressure compared to chimpanzees living in traditional captive settings like research laboratories and zoological institutions—differences that are exacerbated with age (Atencia et al. 2017; Cole et al. 2020; Curry et al. 2023) (but see Renfro et al. 2025). Indeed, the high rates of obesity and associated morbidity (e.g., metabolic syndrome, chronic renal disease) that afflict laboratory chimpanzees (Clay et al. 2022; Nunamaker et al. 2012; Pontzer 2023; Videan et al. 2007) are largely absent in African sanctuaries. Sanctuary chimpanzees also show overall slower rates of biological aging as indexed by a multi‐system metric of ‘physiological dysregulation’ accounting for variation across a suite of multiple biomarkers of health (Cole et al. 2024). Current data on inflammation specifically also suggest key differences between apes living in different contexts. For example, CRP is clinically elevated in research laboratories (4.4–9.7 mg/L on average) (Ely et al. 2013; Lamperez and Rowell 2005; Videan et al. 2009), but not necessarily in African sanctuaries (0.53–2.32 mg/L on average) (Ronke et al. 2015). IL6 is likewise elevated (> 4.64 pg/mL (Pearson et al. 2003)) in laboratories, especially among obese chimpanzees (MD Anderson, 5 pg/mL for non‐obese individuals and 25 pg/mL for obese individuals (Nehete et al. 2014)). Yet African sanctuary apes also experience some species‐typical immune challenges associated with living in naturalistic environments, unlike laboratory populations (Dunay, Owens, et al. 2023; Dunay, Rukundo, et al. 2023). Accordingly, they display more pro‐inflammatory profiles as they age—including significant increases of neutrophil counts, neutrophil to leukocyte ratios, and platelet microparticle counts, and declines in white blood cell counts and platelet counts (Obanda et al. 2014). Furthermore, dysregulation of the immune system progresses more rapidly for sanctuary than laboratory chimpanzees (Cole et al. 2024). This is consistent with the proposition that recurring infectious exposure accelerates aging of the innate immune system (Fulop et al. 2018; Reyes et al. 2023; Santoro et al. 2021).

One hindrance to this comparative perspective on inflammation is that routine blood collection is not possible among many wild primates, including chimpanzees. This prevents direct comparisons of the serum‐derived biomarkers most commonly studied in humans. Instead, long‐term monitoring of immune function is typically conducted using noninvasive urine sampling in wild and free‐ranging contexts (Cooper et al. 2022; Lucore et al. 2022; Thompson González et al. 2020; Wu et al. 2018). Most work has focused on urinary neopterin, a byproduct of macrophage activity and clinical marker of cellular immune system activation (Eisenhut 2013; Higham et al. 2015). Yet investigations of aging patterns have yielded inconsistent results. Neopterin is generally reported to increase with age in wild and semi‐free‐ranging monkeys (Cooper et al. 2022; Dibakou et al. 2020; Lucore et al. 2022; Müller et al. 2017; Schneider‐Crease et al. 2022). Neopterin levels also increased with age in a cross‐sectional sample of wild chimpanzees from the Ngogo community in Kibale National Park, Uganda (Negrey et al. 2021), but not in a longitudinal study of the neighboring community of Kanyawara (Thompson González et al. 2020). These results may be complicated by the fact that neopterin is highly responsive to the acute response to disease (Eisenhut 2013) and older individuals are more often sick (Coe et al. 2012; Monto 2002). Recent efforts have incorporated additional inflammatory biomarkers accessible in urine (Georgiev et al. 2015; Higham et al. 2020; Müller et al. 2017). For example, immune activation and chronic systemic inflammation can also be indexed using soluble urokinase plasminogen activator receptor (suPAR) (Desmedt et al. 2017; Higham et al. 2020). Oxidative stress can be assessed using a suite of biomarkers, including 8‐hydroxydeoxyguanosine (OHdG; DNA damage) and isoprostane compounds (lipid damage) (Georgiev et al. 2015; Krief et al. 2023; Thompson González et al. 2020). Several studies provide biological validations of these measures, showing that they are responsive to acute infection or severe wounding in wild chimpanzees (Thompson González et al. 2020; Wu et al. 2018). But given variable patterns of response to senescence, illness, and injury (Thompson González et al. 2020), multiple biomarkers prove more informative than just one in assessing inflammatory processes in naturalistic context.

In this study, we aimed to evaluate whether chronic accumulation of inflammation and oxidative stress is characteristic of aging in chimpanzees' natural contexts. To do so, we sampled multiple measures of inflammation and oxidative stress in both wild chimpanzees, for which only urinary sample collection is possible, and semi‐free‐ranging chimpanzees living in two forested African sanctuaries, a context where blood can also be collected during routine health exams (see Figure 1 for availability for each medium). We also qualitatively compared serum biomarkers in the sanctuaries to published values from laboratory chimpanzees to contrast this more naturalistic context to chimpanzees experiencing a more processed diet and greater space restrictions. Together, these comparisons allow us to test the prediction that wild and semi‐free‐ranging environments are associated with reduced inflammation and a blunted inflammaging pattern compared with captivity in chimpanzees, analogous to the effects of industrialized environments on modern human inflammation. If such lifestyle factors are dose‐dependent, we would expect higher inflammation, especially during aging, in sanctuary chimpanzees whose diets are supplemented with small amounts of processed foods and whose ranging patterns are within forested enclosures, compared to the wild where animals feed on wild foods and whose ranging is not directly managed. On the other hand, if increased pathogen exposure contributes to chronic inflammation, we would expect relatively high levels of general and age‐associated inflammation in the more naturalistic settings—especially in the wild, where medical intervention is unavailable.

## 2 Materials and Methods

### 2.1 Ethics Statement

Research at Ngamba Island Chimpanzee Sanctuary and Kanyawara in Uganda was approved by the Uganda Wildlife Authority and the Uganda National Council for Science and Technology; research at Tchimpounga Chimpanzee Sanctuary in the Republic of the Congo was approved by the Ministry of Scientific Research and Technological Innovation in Congo and the Jane Goodall Institute. Work was also approved by the Institutional Animal Care and Use Committees at Harvard University, the University of Michigan, the University of New Mexico, and Tufts University. Research practices and animal care procedures complied with the Pan‐African Sanctuary Alliance standards, and shipment of chimpanzee blood complied with international CITES regulations. Data collected for this project are available from the Dryad Digital Repository: 10.5061/dryad.v15dv429v.

### 2.2 Sanctuary Chimpanzee Data

We assayed biomarkers of inflammation and oxidative stress from chimpanzees at Ngamba Island Chimpanzee Sanctuary and Tchimpounga Chimpanzee Sanctuary. Most sanctuary chimpanzees are wild‐born orphans who were mother‐reared in the wild for 1–3 years and integrated into species‐typical social groups upon arrival at the sanctuary (four subjects in this study were born at the sanctuary due to contraception failure). Age in wild‐born sanctuary chimpanzees was estimated by sanctuary veterinarians on arrival and validated by patterns of dental emergence and body weight during subsequent health checks as possible (Cole et al. 2020; Wobber et al. 2010). Sanctuary chimpanzees in these populations typically sleep in secure indoor quarters and are released during the day into 15–95 ha of tropical forest where they can forage on wild foods. Like U.S. laboratories, Pan‐African sanctuaries utilize a dietary guideline established by the National Research Council (National Research Council 2003; Pan African Sanctuary Alliance 2009), but individual caloric and nutritional intake is not strictly controlled in the way they are in a laboratory context, as animals are provisioned and eat within natural social groups. Specifically, sanctuary chimpanzee diets are supplemented with species‐appropriate fruits and vegetables that vary based on seasonal availability, such as bananas, oranges, apples, papayas, cabbages, carrots, sweet potatoes, and various leafy greens; they can further feed on wild foods in their forest habitats *ad libitum*. They also receive limited amounts of processed foods (such as cornmeal or millet porridge), but not the high‐calorie primate chow that predominates captive chimpanzee diets. Because the forested portion of the sanctuaries is accessible only to the chimpanzees for safety reasons, it is not possible to estimate precise measures of diet composition or physical activity. However, these clearly differ substantially from the biomedical laboratories both in terms of the large forested space access and the known categories of food that are available or provided, and overall more closely approximate conditions of chimpanzees in the wild than laboratory contexts. Finally, while sanctuary chimpanzees show a normal complement of species‐typical (apathogeneic) viruses (Dunay, Owens, et al. 2023; Dunay, Rukundo, et al. 2023), in contrast to wild populations, they also receive routine veterinary care to treat wounds and infection, administer vaccinations, and otherwise assess the health and wellbeing of these animals (Stokes et al. 2018).

Biological samples were collected during these routine health checks conducted by sanctuary veterinarians at Tchimpounga (*N* = 26 individuals) in 2019 and Ngamba (*N* = 47 individuals) in 2016, 2017, and 2023 (see Figure S1 and Table 1 for summary of age distributions and sample sizes for each biomarker by site). Given our focus on aging, we excluded immature chimpanzees younger than 10 years old (Pusey 1990). Chimpanzees are typically considered to be young adults between 15 and 20 years old, in prime adulthood from 20 to 35 years, and ‘old’ after 35 years; wild chimpanzees in their 30s show physical and behavioral signatures of aging as well as increased mortality (Emery Thompson, Fox, et al. 2020; Emery Thompson, Machanda, et al. 2020; Hill et al. 2001; Rosati et al. 2020). As we were interested in patterns of normal aging as opposed to acute responses to infection, we also excluded data from chimpanzees who were very ill at the time of sampling (i.e., where notes indicated they required intervention, or where out‐of‐range results for several biomarkers indicated severe illness at the time of sample collection; *N* = 4 datapoints from 4 chimpanzees). After these exclusions, the full sanctuary dataset included 156 health check timepoints from 36 females and 37 males ranging from 11.1 to 39.0 years (mean age = 22.3 years). Blood at both sites was collected by veterinarians via venipuncture into EDTA‐coated tubes to prevent coagulation. Urine samples at both sites were collected into sterile vials via manual expression or catheterization. If we did not obtain sufficient sample during the health check, we collected fresh samples within 24 h by catching urine directly in vials tied to a long stick with wire, or in large plastic specimen bags, from chimpanzees held in overhead raceways of nighttime dormitories. All samples were refrigerated upon collection and processed within several hours. We centrifuged EDTA tubes at 3500 RPM for 10 min to separate the serum layer. Serum and urine were aliquoted into minitubes using disposable transfer pipettes and stored frozen until analysis.

**TABLE 1:** Summary of sample sizes for each biomarker by site.

	Ngamba (sanctuary)	Tchimpounga (sanctuary)	Kanyawara (wild)
Total	130 time points 47 individuals (28 females, 10 juveniles, 39 adults, 16 older adults)	26 time points 26 individuals (8 females, 1 juvenile, 25 adults, 0 older adults)	1849 time points 50 individuals (28 females, 28 juveniles, 37 adults, 16 older adults)
uNeopterin	129 samples (2016, 2017, 2023)	23 samples (2019)	1681 samples (2008–2024)
usuPAR	42 samples (2023)	—	581 samples (2013–2024)
uIsoprostanes	123 samples (2016, 2017, 2023)	24 samples (2019)	1303 samples (2008–2024)
uOHdG	129 samples (2016, 2017, 2023)	22 samples (2019)	1819 samples (2008–2024)
sCRP	84 samples (2016, 2017)	23 samples (2019)	—
sIL6	87 samples (2016, 2017)	20 samples (2019)	—

*Note:* We classified juveniles as 10–15 years, adults as 15–30 years, and older adults as over 30, following prior work (Cole et al. 2020; Hill et al. 2001). Note that the same individual could appear in multiple age categories throughout the study. usuPAR was not available at Tchimpounga and blood markers were not available at Kanyawara. This represents the final dataset after sample exclusions defined in the text.

### 2.3 Wild Chimpanzee Data

We compared urinary results to those from the Kanyawara community of wild chimpanzees, who reside in Kibale National Park in western Uganda. Kanyawara chimpanzees live in a ~10 km^2^ home range of mosaic rainforest, traveling an average of 2.1 km per day (Chapman and Wrangham 1993; Emery Thompson, Muller, et al. 2020; Pontzer and Wrangham 2006; Rutherford 2021). They primarily feed on ripe drupe fruits, figs, and herbaceous pith, but a small proportion of the diet is made up of animal protein, domesticated crops from nearby villages, and honey (Emery Thompson, Muller, et al. 2020). Adult females consume an estimated 1240–4931 kcal/day (Uwimbabazi et al. 2019). Urine sampling has been conducted continuously at Kanyawara since 1998 for longitudinal monitoring of chimpanzee health and physiology. Our general strategy for this study was to analyze one sample per individual on a quarterly basis, preferentially from first morning voids, as the sample availability allowed. We included no more than one sample per chimpanzee per day. The Kanyawara dataset spanned 2008–2024 and totaled 1849 sampling timepoints from 50 adult chimpanzees (*N* = 28 females) ranging from 10.0 to 57.8 years (mean age = 25.7 years; see Figure S1 for a summary of age distribution and sample sizes for each biomarker). Samples collected during respiratory disease outbreaks (4 months in 2013 and 1 month in 2017) were excluded from this analysis. Assay methods for the Kanyawara sample are also described in detail in (Thompson González et al. 2020), which provided results for 2008–2017.

### 2.4 Assay Methods

We assayed all samples at the University of New Mexico Comparative Human and Primate Physiology Center. We measured C‐reactive protein (sCRP, Catalog No. KHA0031, Thermo Fisher Scientific) and interleukin‐6 (sIL6, Catalog No. BMS213HS, Thermo Fisher Scientific) in serum samples, which were only available from sanctuary chimpanzees. We excluded samples outside of assay detection ranges, that is, ‘spikes’ that indicate active infection. All samples were run in duplicate, and we excluded serum results with a coefficient of variation (CV) over 25%. After these exclusions, intra‐assay CVs were 3.3% for sCRP and 6.2% for sIL6. Inter‐assay CVs for high and low controls were 10.9% and 12.8% for sCRP; and 10.2% and 4.9% for sIL6. All serum results were log transformed to approach normality. Specific assay methods for sCRP and sIL6 in our study sometimes varied from the various published reports to which we compare them. However, sCRP and sIL6 kits are calibrated to human clinical reference thresholds, suggesting they should be broadly comparable across studies.

All urinary biomarkers were assessed using identical protocols for the sanctuaries and wild Kanyawara chimpanzees. Dilute samples were excluded from analysis (specific gravity below 1.003 at Kanyawara or below 1.002 at the sanctuaries following (Cole et al. 2024)). We measured neopterin (uNeopterin, Ref. RE59321, IBL International), soluble urokinase plasminogen activator receptor (usuPAR, Catalog No. ELH‐uPAR, Ray Biotech), 15‐Isoprostane F_2t_ (uIsoprostanes, Prod. No. E85, Oxford Biomedical Research), and 8‐hydroxy‐2′‐deoxyguanosine (uOHdG, Catalog No. KOG‐200S/E, Genox JaICA). Average recovery of usuPAR enzyme immunoassays on spiked samples was 89% (54%–90%). Using the same kits, prior studies reported 100.3% recovery of uNeopterin, 114% recovery of uIsoprostanes (106%–123%), and 116% recovery of uOHdG (88%–137%) from spiked chimpanzee urine samples (Behringer et al. 2017; Thompson González et al. 2020).

For these urinary assays, some results were outside the assay detection limit. Most of these were results that were under the lower limit of quantification, in which case we assigned them a minimum value for that assay (250 nmol/L for uNeopterin, 8% of results; 75 pg/mL for usuPAR, 6% of results; 0.2 ng/mL for uIsoprostanes, < 1% of results; and 0.5 ng/mL for uOHdG, 2% of results) (Aronoff et al. 2025). Results over the upper limit of quantification were assigned a maximum value for that assay (4375 nmol/L for uNeopterin, 3% of results; 20,000 pg/mL for usuPAR, 1% of results; 400 ng/mL for uIsoprostanes, < 1% of results; and 200 ng/mL for uOHdG, < 1% of results). For samples within the assay detection range, we excluded all results with CVs over 50%. CVs above 25% were allowed only for results close to the lower detection limit (less than 500 nmol/L for uNeopterin; 400 pg/mL for usuPAR; 2 ng/mL for uIsoprostanes; and 1 ng/mL for uOHdG). Intra‐assay CVs for samples within detection ranges were 5.6% for uNeopterin; 5.6% for usuPAR; 5.9% for uIsoprostanes; and 6.1% for uOHdG. Inter‐assay CVs for high and low controls were 9.3% and 18.8% for uNeopterin; 7.6% and 12.2% for usuPAR; 14.3% and 16.7% for uIsoprostanes; and 10.6% and 16.1% for uOHdG. All urinary results were corrected for specific gravity (relative to a population mean of 1.017) and log transformed to approach normality. Published uNeopterin reports from different wild sites use the same assay reagents and protocols but were analyzed in different labs. These studies also used a comparable population mean for specific gravity correction (1.017 in Wu et al. 2018; 1.018 in Negrey et al. 2021).

### 2.5 Statistical Analyses

We first examined the relationship among all available biomarkers at the sanctuaries and Kanyawara using pairwise Pearson's correlations. While these biomarkers correspond to different physiological processes related to the pathways of inflammation, we expected them to be positively correlated. Importantly, this allowed us to validate the assumption that the non‐invasive measures from urine commonly used with wild populations are in fact related to blood‐derived measures and therefore can be used to make broader inferences about systemic inflammation in wild primates.

We then compared biomarker levels across ages and living contexts. We focused mainly on a direct comparison between the sanctuaries and Kanyawara using linear mixed effects models and the *lmer* function in R. All models included a random effect of *subject identity* to account for unbalanced repeated measurements across multiple timepoints and *site year* and *assay year* to account for additional random variation. Specifically, *site year* refers to the location and year of sample collection (21 levels), while *assay year* refers to year of sample analysis (five levels). Our general approach was to first construct a base model for each urinary biomarker (uNeopterin, usuPAR, uIsoprostanes, and uOHdG), which included fixed effects for *time of day* and *sex* (female or male) following prior work (Negrey et al. 2021; Thompson González et al. 2020). *Time of day* was z‐scored to approximate the scale of other predictor variables. To test for age‐related increases in biomarker concentrations, we then used likelihood ratio tests to assess whether including *age* improved model fit. Exploratory analyses showed that *age* ^2^ and age*sex terms were never significant, so they were not included in our formal analyses. We subsequently tested if overall levels or age‐related changes in biomarker levels varied between populations, that is, whether model fit was further improved by including *facility* (sanctuary or wild) or age*facility, respectively.

We also constructed separate models for the sanctuary and wild datasets to assess potentially unique age effects in each population. For sanctuary chimpanzees, we used the same approach for urinary and serum markers, except that sera analyses did not control for time of day. We additionally controlled for sanctuary *site* (Ngamba or Tchimpounga) in sanctuary models. We did not include the random effect of *assay year* for the sanctuary or usuPAR models, since these assays were all run within 2 years. For sanctuary usuPAR, which was only available for one set of health checks at Ngamba, we used a linear model implemented with the *lm* function.

## 3 Results

### 3.1 Correlations Across Urinary and Serum Markers

In a combined dataset including Kanyawara and the sanctuaries, pairwise Pearson correlations showed significant positive correlations among all urinary markers except between usuPAR and uOHdG (see Table 2 for details). At the sanctuaries, where both urine and serum markers were available, neither sCRP nor sIL6 were significantly correlated with any of the other biomarkers. This may be related to the small sample size at the sanctuaries given that urinary markers were highly correlated in the wild population alone but showed weaker relationships in the sanctuaries (see Tables S1 and S2 for correlations in the sanctuary and wild datasets, respectively).

**TABLE 2:** Correlations among biomarkers in sanctuary and wild chimpanzees.

	usuPAR	uIsoprostanes	uOHdG	sCRP	sIL6
uNeopterin	*R* = 0.42***	*R* = 0.26***	*R* = 0.17***	*R* = −0.07	*R* = 0.17.
usuPAR		*R* = 0.34***	*R* = 0.07.	*NA*	*NA*
uIsop			*R* = 0.20***	*R* = −0.03	*R* = 0.15
uOHdG				*R* = −0.14	*R* = 0.06
sCRP					*R* = 0.14

*Note:* Urinary markers are corrected for specific gravity and all markers are log transformed. Correlations between urinary markers involve both wild and sanctuary data, whereas correlations involving serum markers only come from sanctuary populations where these were available. There were no matching serum samples to compare with urinary suPAR measures. Asterisks denote significance. ***p < 0.001; **p < 0.01; *p < 0.05; *p* < 0.10.

### 3.2 Urinary Markers in Sanctuary and Wild Chimpanzees

We first assessed the effects of aging on urinary marker concentrations in a combined dataset including chimpanzees from Ngamba, Tchimpounga, and Kanyawara (see data distributions for each biomarker in Figure S2). Including *age* did not improve model fit relative to the base model (which included only time of sampling, sex, and random effects) for uNeopterin [χ2 = 0.37; d.f. = 1; *p* = 0.54], uIsoprostanes [χ2 = 0.44; d.f. = 1; *p* = 0.50], or uOHdG [χ2 = 0.36; d.f. = 1; *p* = 0.55]. However, we detected a significant and positive age effect for usuPAR [χ2 = 4.75; d.f. = 1; *p* = 0.03]. Compared to females, males had higher levels of usuPAR (ß = 0.32; *p* = 0.02) and uIsoprostanes (ß = 0.16; *p* < 0.001), lower levels of uOHdG (ß = −0.24; *p* < 0.001), and equivalent levels of uNeopterin.

**FIGURE 2:**
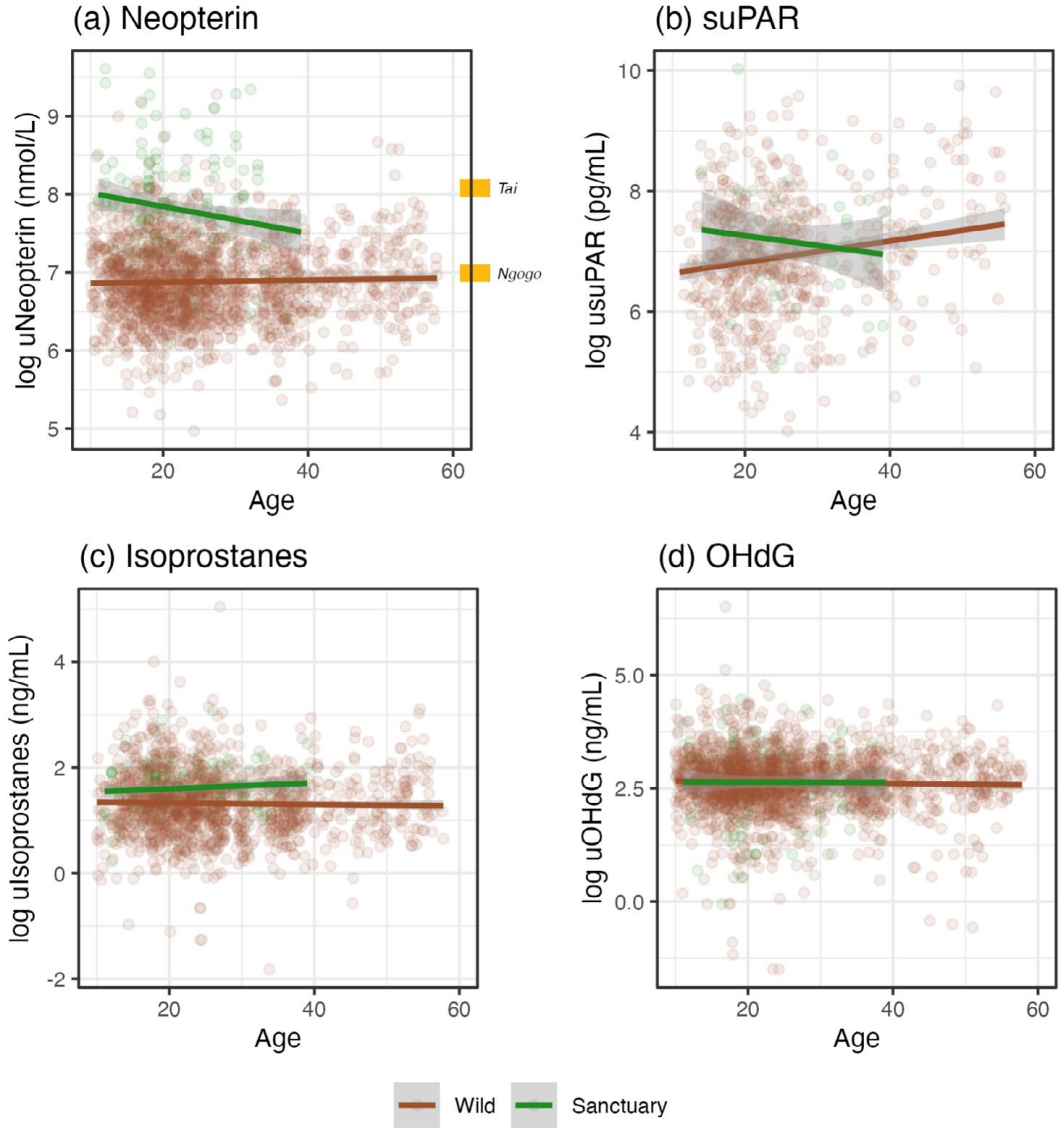
Age‐related change in urinary biomarkers. African sanctuary and wild chimpanzee (Kanyawara) comparisons of (a) uNeopterin, (b) usuPAR, (c) uIsoprostanes, and (d) uOHdG; lines indicate linear fit; ribbons indicate 95% confidence intervals. Urinary markers are corrected for specific gravity. For uNeopterin, gold references are published average values from two other wild chimpanzee sites: Ngogo in Uganda (1081 nmol/L (Negrey et al. 2021)) and Taï in Ivory Coast (3220 nmol/L (Wu et al. 2018)).

We then examined whether biomarker levels and their age‐related changes differed between sanctuary and wild chimpanzees (Figure 2; see Table S3 for model results). uNeopterin was higher at the sanctuaries (mean = 3139 nmol/L) than at Kanyawara (mean = 1119 nmol/L) [χ2 = 30.37; d.f. = 1; *p* < 0.001]. We found the same pattern for uIsoprostanes [sanctuary mean = 5.48 ng/mL; Kanyawara mean = 4.71 ng/mL; χ2 = 13.64; d.f. = 1; *p* < 0.001]. Meanwhile, usuPAR levels were not statistically different at the sanctuaries (mean = 2106 pg/mL) and at Kanyawara (mean = 1653 pg/mL) [χ2 = 3.74; d.f. = 1; *p* = 0.05]. We also found no difference for uOHdG by facility [sanctuary mean = 18.26 ng/mL; Kanyawara mean = 17.26 ng/mL; χ2 = 0.01; d.f. = 1; *p* = 0.92]. We did not detect significant age*facility effects for any biomarker, indicating that the aging effect did not differ between the wild and sanctuary populations.

We repeated these models for sanctuary and wild populations separately (see Tables S4 and S5 for results). These models confirmed that the two sanctuaries did not systematically differ from one another. Consistent with our main, combined analysis, the only finding for age‐related increase was for usuPAR, but this was statistically significant only in the wild Kanyawara sample. Because the effect size of aging was very weak (ß = 0.01 in the combined sample and ß = 0.02 in the Kanyawara sample) and did not differ significantly between environments in our main analysis, we suspect this difference is due to insufficient power in the sanctuary‐only sample. As shown in Figure 2, even the oldest chimpanzees in the Kanyawara sample fall within the confidence interval of the sanctuary sample.

### 3.3 Serum Markers in Sanctuary and Laboratory Chimpanzees

**FIGURE 3:**
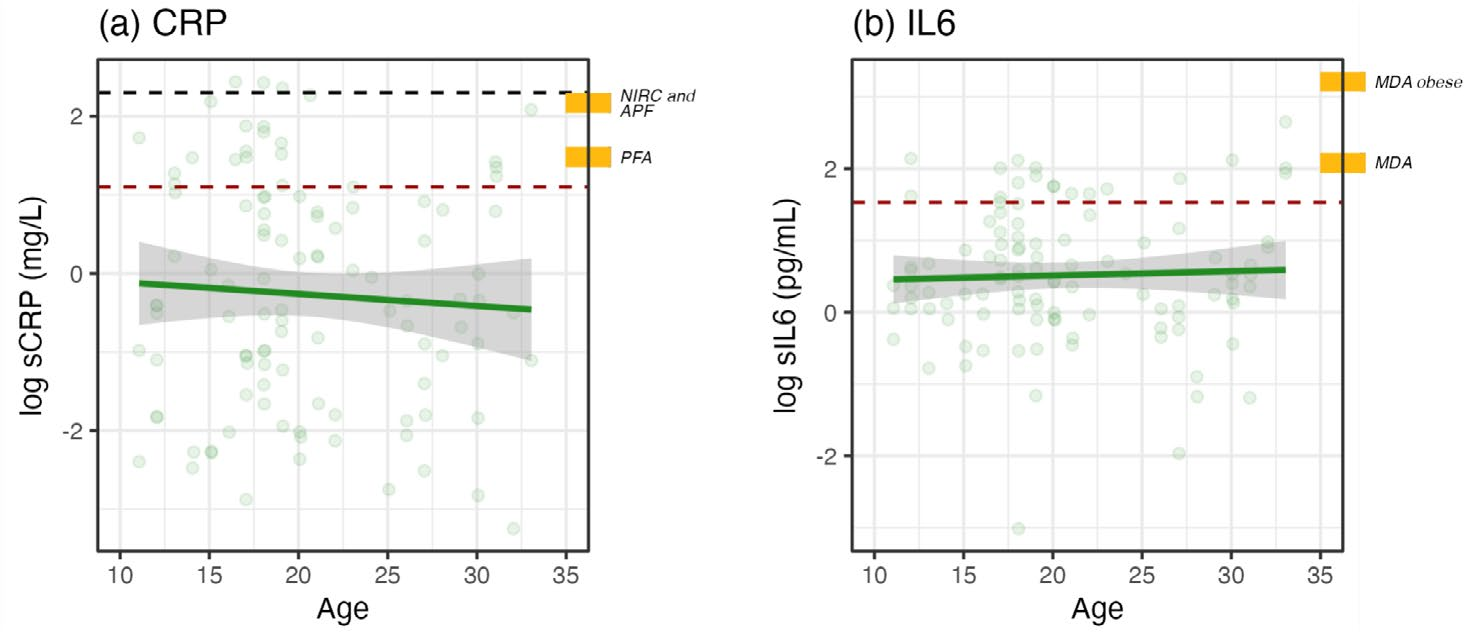
Age‐related change in serum biomarkers. African sanctuary chimpanzee values for (a) sCRP and (b) sIL6; lines indicate linear fit; ribbons indicate 95% confidence intervals. In both panels, gold references are previously published average values from US laboratory chimpanzees. For sCRP, PFA = Primate Foundation of Arizona (4.4 mg/L (Videan et al. 2009)); APF = Alamogordo Primate Facility (8 mg/L (Ely et al. 2013)); and NIRC = New Iberia Research Center (9.7 mg/L (Lamperez and Rowell 2005)). For sIL6, MDA = MD Anderson Cancer Center (5 pg/mL for non‐obese chimpanzees; 25 pg/mL for obese chimpanzees (Nehete et al. 2014)). Red dashed lines indicate human cutoffs for clinical elevation (over 3 mg/L for sCRP and over 4.64 pg/mL for sIL6). Black dashed line indicates human cutoff for acute infection (over 10 mg/L for sCRP). Risk thresholds are from Pearson et al. 2003.

We finally examined the serum markers, which were only available from sanctuary chimpanzees (see data distributions for each biomarker in Figure S3). We detected no significant effect of age for sCRP or sIL6 (Figure 3; see Table S4 for model results). sIL6, but not sCRP, was higher for males than females (ß = 0.56; *p* < 0.01) and higher at Tchimpounga than Ngamba (ß = 0.71; *p* < 0.01). Sanctuary CRP and IL6 generally fell within ‘normal’ human ranges as defined by the US Centers for Disease Control (Pearson et al. 2003) (Figure 3). Mean CRP was 1.89 mg/L; 84/107 samples (79%) were lower than the clinically elevated human threshold of 3 mg/L, and 104/107 samples (97%) were below the threshold for acute infection of 10 mg/L (Pearson et al. 2003). Mean IL6 was 2.47 pg/mL; 89/107 samples (83%) fell below the elevated human threshold of 4.64 pg/mL (Pearson et al. 2003).

## 4 Discussion

In this study, we assessed inflammatory profiles across adulthood in one of human's closest living relatives, the chimpanzee. To address the challenge of obtaining the most commonly used blood biomarkers of inflammation from wild chimpanzees, we obtained these measures from wild‐born, semi‐free‐ranging chimpanzees living in African sanctuaries. Unlike in industrialized human populations (Ferrucci and Fabbri 2018; López‐Otín et al. 2023; Pearson et al. 2003; Pizzino et al. 2017), C‐reactive protein (CRP) and interleukin‐6 (IL6) were generally below the threshold for clinical elevation and did not increase significantly with age over this multi‐year study. We also did not detect a signature of inflammation in our panel of non‐invasive urinary markers of inflammation and oxidative stress in sanctuaries and the wild. While urinary suPAR did increase significantly with age, the effect size was very small and statistically significant only in the wild sample, which had both a larger sample size and an older age range than the sanctuary sample. The unique combination of a naturalistic lifestyle and routine blood sampling situates chimpanzees living in African sanctuaries as a tractable evolutionary model for the human inflammaging process. Despite experiencing sometimes extreme early life stress, adult environments appear to buffer against chronic inflammation and other negative age‐related outcomes typical of industrial human contexts. As chimpanzee sanctuary populations age, longitudinal sampling will be crucial to test the extent to which this physiological resilience persists into the period of senescence, and how it affects patterns of mortality. More broadly, our approach of leveraging overlapping physiological datasets from a range of living environments can shed light on the nuanced ways in which inflammatory processes respond to environmental input across the life course.

As in prior research from wild chimpanzees living in the Kanyawara community (Thompson González et al. 2020), urinary measures of immune activation, inflammation, and oxidative stress were positively correlated, both at this wild site and in analyses including the sanctuary chimpanzee data. Given the limitations surrounding blood collection in wild primates, we were particularly interested in whether these non‐invasive markers correlated with circulating markers of inflammation obtained from serum. However, neither CRP nor IL6 levels were significantly correlated with any of the urinary biomarkers. We suspect this to be in part because, outside of a few instances of severe illness where serum readings were too high to be quantified and were excluded, levels were maintained at such a low level in this population that variation may not be biologically significant. As urinary suPAR—a marker more strongly associated with chronic inflammation in other research (Desmedt et al. 2017; Thunø et al. 2009)—was introduced late in the study when there were no matching blood samples available to assay, we were unable to evaluate its correlation to the serum biomarkers, which would be an important focus for future work.

Semi‐free‐ranging African sanctuary chimpanzees maintained low levels of inflammation compared to previously published reports from more sedentary chimpanzees living in laboratories. Mean CRP (1.89 mg/L) was lower than previously published averages from laboratory chimpanzees living at Primate Foundation of Arizona (4.4 mg/L (Videan et al. 2009)), Alamogordo Primate Facility (8 mg/L (Ely et al. 2013)), and New Iberia Research Center (9.7 mg/L (Lamperez and Rowell 2005)), and more similar to previously published reports from African sanctuary populations, specifically Tchimpounga (2.32 mg/L) and Tacugama Chimpanzee Sanctuary in Sierra Leone (0.53 mg/L) (Ronke et al. 2015) (Figure 3). Mean IL6 (2.47 pg/mL) was about 2–10 times lower than a prior study of laboratory chimpanzees at MD Anderson (5 pg/mL for non‐obese chimpanzees and 25 pg/mL for obese individuals (Nehete et al. 2014)). While it is more difficult to compare absolute concentrations of IL6 across assays, these differences are dramatic enough to suggest a true physiological difference, as opposed to an artifact of variance in assay sensitivity. We note that while the sanctuary populations experience qualitatively different living conditions from many laboratory‐living populations in terms of their overall diet and space access, we did not aim to precisely characterize individual variation in their consumption patterns or physical activity. Such detailed observations could contextualize individual variation in chimpanzee biomarkers in future work, in line with experimental findings linking specific diets and caloric levels to inflammation markers and overall health in other primate species (Mattison et al. 2017; Wood and Sullivan 2022). More generally, the current findings align with prior evidence from chimpanzees indicating that the impact of diet and exercise in promoting healthy aging in humans is shared with other apes (Atencia et al. 2017; Cole et al. 2020, 2024; Curry et al. 2023).

Two of four urinary biomarkers were higher in the sanctuary populations compared to our wild sample: neopterin and isoprostanes. We do not know whether these differences are of a magnitude that may be consequential for health. For example, sanctuary neopterin levels were within the previously reported range of normal variation for wild chimpanzees at Ngogo (mean = 1081 nmol/L (Negrey et al. 2021)), and very similar on average to a ‘healthy’ (pre‐respiratory outbreak) period in the wild Taï community (mean = 3220 nmol/L (Wu et al. 2018)) (Figure 2). A further important question is what drives these site differences. One possibility is that semi‐free‐ranging chimpanzees have higher energy balance to devote to the immune system at baseline, given that they experience higher, or at least more stable, caloric and nutrient intakes than their wild counterparts due to active provisioning. They may also experience fewer energetic demands, such as pregnancy and lactation, because females are typically on birth control. This would be consistent with higher neopterin in zoo versus wild chimpanzees (Behringer et al. 2017) and higher neopterin with higher energy balance in developing guenons (Thompson González et al. 2025). Alternatively, higher inflammation and oxidative stress could also potentially reflect increased pathogen burden at the sanctuaries. While we did not directly measure infection in the current study, prior work has shown that sanctuary chimpanzees exhibit a viral metagenomic profile that largely resembles those of their wild counterparts, and are moreover largely apathogenic (Dunay, Owens, et al. 2023; Dunay, Rukundo, et al. 2023). Since sanctuary chimpanzees are often de‐wormed during routine health checks, and treated for illness as needed, they may in fact experience lower *severity* of infection relative to wild communities, where respiratory disease in particular is a leading cause of morbidity and mortality (Emery Thompson et al. 2018; Gurven and Gomes 2017; Negrey et al. 2019). However, sanctuary chimpanzees are also more densely populated compared to their wild counterparts, which could increase the spread of disease (Griffin and Nunn 2012; Hu et al. 2013; Rosati, Sabbi, et al. 2023). A final consideration is that sanctuary chimpanzees may have more chronic inflammation. Most individuals arrive at the sanctuary early in development as victims of the bushmeat and pet trade, and may be malnourished or sick from these early experiences (Pan African Sanctuary Alliance 2009). Although these adverse early environments do not appear to drive increased lifelong inflammation, they may contribute to some overall dysregulation of the immune system (Cole et al. 2024).

The other two urinary markers, suPAR and OHdG, did not statistically differ between Kanyawara and the sanctuaries. OHdG can be particularly challenging to interpret as it reflects both DNA damage and repair, mechanisms that may progress differentially with age in long‐lived primates (Georgiev et al. 2015; Thompson González et al. 2020). However, prior work in chimpanzees has demonstrated that OHdG increases with exposure to agricultural pesticides and also increases with a drastic decline in dietary antioxidants (Costantini et al. 2021; Krief et al. 2023). While dietary composition was not measured here, higher levels of antioxidants in a partially domesticated diet could be expected to improve OHdG status among sanctuary animals. Detailed information on caloric, nutritional, and pesticide intake will be key to future studies of inflammation and related processes.

Overall, our results indicate that the sanctuary and wild populations exhibit both some commonalities and some differences in the suite of urinary biomarkers we measured. While it is difficult to interpret why some biomarkers vary between these environments and others do not, work in wild chimpanzees suggests that urinary neopterin, OHdG, and isoprostanes can show differing magnitudes and timing of response to different types of health challenges (Thompson González et al. 2020). Additionally, some markers, such as suPAR, have been reported to provide a stronger signal of chronic versus acute inflammation (Desmedt et al. 2017; Thunø et al. 2009). Given the lower levels of comparable inflammatory markers in sanctuaries versus laboratories, the fact that wild chimpanzees had lower concentrations than sanctuary chimpanzees for some of these markers suggests that general and age‐related inflammation is at a minimum in this wild setting.

Importantly, we did not find evidence of a clear immunoaging phenotype in either the sanctuary or wild populations, supporting prior conclusions based on a subset of the current Kanyawara dataset (Thompson González et al. 2020). This is different from a recent report of a modest but significant age effect on urinary neopterin from Ngogo (Negrey et al. 2021). The Ngogo sample included a larger number of chimpanzees of extreme old age (up to age 67) than did either the wild or sanctuary datasets analyzed here. Still, these results are striking given that the Ngogo community is a part of the same contiguous population as Kanyawara, located only approximately 12 km away. The Ngogo chimpanzee community has a higher density of fruiting trees (Potts et al. 2011) and higher energy balance (Emery Thompson et al. 2009) than Kanyawara, as well as lower loads of viruses (Negrey et al. 2022) and parasites (Phillips et al. 2020)—conditions that should predict a relatively low level of inflammation. Our comparative examination helps put these age increases in perspective, as even the oldest Ngogo chimpanzees appear to have neopterin concentrations at the low end of the variation in chimpanzees. We also note that our prior results on dysregulation in sanctuary chimpanzees (Cole et al. 2024) indicated that dysregulation of the immune system proceeded more rapidly at Ngamba compared to U.S. laboratories, suggesting faster immunological aging in a population facing more natural patterns of infections. This discrepancy could be because, while the sanctuary dataset in Cole et al. (2024) included the serum data we used here, it also included additional measures derived from white blood cell counts, such as neutrophil to lymphocyte ratio (NLR), a common inflammatory disease risk factor in humans that is reported to increase with age at a Kenyan sanctuary (Obanda et al. 2014).

More broadly, the contrast between these results are suggestive of a subtle distinction between proinflammatory pathways of a senescent immune system, exhausted by persistent immune challenges, and the more adverse ‘inflammaging’ pathway, where lifestyle risk factors like obesity and stress both increase chronic inflammation and accelerate immunosenescence (Fulop et al. 2018; Santoro et al. 2021). Captive facilities often maintain sterile environments, so laboratory chimpanzees likely experience relatively little exposure to infectious disease and associated immunosenescence. More naturalistic environments, on the other hand, are rife with immunological challenges, and there is abundant evidence that wild chimpanzees are more vulnerable to illness and parasitism as they age (Emery Thompson et al. 2018; Negrey et al. 2019, 2020; Phillips et al. 2020). Indeed, neopterin and isoprostane concentrations increased in the years of declining health preceding death in past‐prime individuals at Kanyawara (Thompson González et al. 2020).

While the current study includes a relatively large proportion of older adults (over 30 years) compared with prior reports, these African sanctuary populations were founded only in the 1990s and are still relatively young compared to wild sites. Our sample reaches age 33 for serum markers and age 39 for urinary markers. The nature of this sample limits statistical power, particularly if the effects of chronic inflammation are only evident at late ages. However, our results align with prior work showing no major age‐related shifts in blood lipids, obesity, or physiological dysregulation in sanctuary populations compared to laboratory‐living chimpanzees, even when sampling age ranges are equated (Atencia et al. 2017; Cole et al. 2020, 2024; Curry et al. 2023). Overall, the lack of aging shifts in serum markers in the sanctuary chimpanzees, and the overlapping urinary biomarkers in both the wild and sanctuary chimpanzees, support the hypothesis that increases in chronic inflammation are not an inevitable outcome of the aging process in our close relatives. Paired with evidence that chronic inflammation is reduced or absent in humans in small‐scale subsistence versus industrialized populations, our findings support the hypothesis that inflammaging is not an ancestral process in our species but is a result of a mismatch between our evolved aging biology and novel environments (Eaton et al. 1988; Gurven and Lieberman 2020).

## Author Contributions

**Megan F. Cole:** conceptualization (equal), data curation (lead), formal analysis (lead), investigation (equal), methodology (equal), visualization (lead), writing – original draft (lead), writing – review and editing (lead). **Melissa Emery Thompson:** conceptualization (equal), data curation (equal), funding acquisition (equal), investigation (equal), methodology (equal), supervision (equal), writing – original draft (equal), writing – review and editing (equal). **Nicole Thompson González:** investigation (equal), writing – review and editing (equal). **Eleanor Paskus:** investigation (equal), writing – review and editing (supporting). **Joshua Rukundo:** investigation (equal), writing – review and editing (supporting). **Rebeca Atencia:** investigation (equal), writing – review and editing (supporting). **Alexandra G. Rosati:** conceptualization (equal), data curation (equal), funding acquisition (equal), methodology (equal), project administration (lead), resources (lead), supervision (lead), writing – original draft (equal), writing – review and editing (equal).

## Funding

This study was funded by the National Institute on Aging and the Office of Research on Women's Health of the National Institutes of Health (grants R01‐AG049395, R37‐AG049395, and R61‐AG078468) and the Directorate for Social, Behavioral and Economic Sciences (BCS‐2141766) and a Graduate Research Fellowship (GRFP) to Megan F. Cole.

## Supporting information


**Table S1:** Correlations among biomarkers in sanctuary chimpanzees. Urinary markers are corrected for specific gravity and all markers are log transformed. Asterisks denote significance. *** *p* < 0.001; ** *p* < 0.01; * *p* < 0.05; *p* < 0.10.
**Table S2:** Correlations among biomarkers in wild chimpanzees. Urinary markers are corrected for specific gravity and all markers are log transformed. Asterisks denote significance. ***p < 0.001; **p < 0.01; *p < 0.05; *p* < 0.10.
**Table S3:** Predictors of urinary markers in sanctuary and wild chimpanzees. Markers are corrected for specific gravity and log transformed. Results are from models that exclude the age*facility interaction, since this was never a significant predictor.
**Table S4:** Predictors of urinary and serum markers in sanctuary chimpanzees. Urinary markers are corrected for specific gravity, and all markers are log transformed. Results are from full models.
**Table S5:** Predictors of urinary markers in wild chimpanzees. All markers are corrected for specific gravity and log transformed. Results are from full models.
**Figure S1:** Age distributions of sanctuary versus wild chimpanzees.
**Figure S2:** Histograms of urinary markers in sanctuary versus wild chimpanzees. All markers are corrected for specific gravity and log transformed.
**Figure S3:** Histograms of serum markers in sanctuary chimpanzees. All markers are log transformed. Red dashed lines indicate human cutoffs for clinical elevation (over 3 mg/L for CRP and over 4.64 pg/mL for IL6). Black dashed line indicates human cutoff for acute infection (over 10 mg/L for CRP). Risk thresholds are from (Pearson et al. 2003).

## Data Availability

Data collected for this project are available from the Dryad Digital Repository: 10.5061/dryad.v15dv429v.
