# Supplementary material for: Minimal Evidence of Inflammaging in Naturalistic Chimpanzee Populations
Source: Am J Biol Anthropol. 2026 Feb 9;189(2):e70211. doi: 10.1002/ajpa.70211 (PMC12884218; doi:10.1002/ajpa.70211)
Supplement: Supplementary file 1 — Table S1: Correlations among biomarkers in sanctuary chimpanzees. Urinary markers are corrected for specific gravity and all markers are log transformed. Asterisks denote significance. *** p < 0.001; ** p < 0.01; * p < 0.05; p < 0.10. Table S2: Correlations among biomarkers in wild chimpanzees. Urinary markers are corrected for specific gravity and all markers are log transformed. Asterisks denote significance. ***p < 0.001; **p < 0.01; *p < 0.05; p < 0.10. Table S3: Predictors of urinary markers in sanctuary and wild chimpanzees. Markers are corrected for specific gravity and log transformed. Results are from models that exclude the age*facility interaction, since this was never a significant predictor. Table S4: Predictors of urinary and serum markers in sanctuary chimpanzees. Urinary markers are corrected for specific gravity, and all markers are log transformed. Results are from full models. Table S5: Predictors of urinary markers in wild chimpanzees. All markers are corrected for specific gravity and log transformed. Results are from full models. Figure S1: Age distributions of sanctuary versus wild chimpanzees. Figure S2: Histograms of urinary markers in sanctuary versus wild chimpanzees. All markers are corrected for specific gravity and log transformed. Figure S3: Histograms of serum markers in sanctuary chimpanzees. All markers are log transformed. Red dashed lines indicate human cutoffs for clinical elevation (over 3 mg/L for CRP and over 4.64 pg/mL for IL6). Black dashed line indicates human cutoff for acute infection (over 10 mg/L for CRP). Risk thresholds are from (Pearson et al. 2003). [file AJPA-189-e70211-s001.docx]

**SUPPORTING INFORMATION**

Minimal evidence of inflammaging in naturalistic chimpanzee populations

Megan F. Cole, Melissa Emery Thompson, Nicole Thompson González, Eleanor Paskus, Joshua Rukundo, Rebeca Atencia, & Alexandra G. Rosati

**Table S1. Correlations among biomarkers in sanctuary chimpanzees.** Urinary markers are corrected for specific gravity and all markers are log transformed. Asterisks denote significance. *** p<0.001; ** p< 0.01; * p< 0.05; . p<0.10.

|  | **usuPAR** | **uIsoprostanes** | **uOHdG** | **sCRP** | **sIL6** |
| --- | --- | --- | --- | --- | --- |
| **uNeopterin** | *R* = 0.26 | *R* = 0.14  *.* | *R* = 0.16  . | *R* = -0.07 | *R* = 0.17  . |
| **usuPAR** |  | *R* = 0.23 | *R* = 0.06 | *NA* | *NA* |
| **uIsop** |  |  | *R* = -0.01 | *R* = -0.03 | *R* = 0.15 |
| **uOHdG** |  |  |  | *R* = -0.14 | *R* = 0.06 |
| **sCRP** |  |  |  |  | *R* = 0.14 |

**Table S2. Correlations among biomarkers in wild chimpanzees.** Urinary markers are corrected for specific gravity and all markers are log transformed. Asterisks denote significance. *** p<0.001; ** p< 0.01; * p< 0.05; . p<0.10.

|  | **usuPAR** | **uIsoprostanes** | **uOHdG** | **sCRP** | **sIL6** |
| --- | --- | --- | --- | --- | --- |
| **uNeopterin** | *R* = 0.43  *** | *R* = 0.23  *** | *R* = 0.19  *** | *NA* | *NA* |
| **usuPAR** |  | *R* = 0.34  *** | *R* = 0.07 | *NA* | *NA* |
| **uIsop** |  |  | *R* = 0.22  *** | *NA* | *NA* |
| **uOHdG** |  |  |  | *NA* | *NA* |
| **sCRP** |  |  |  |  | *NA* |

**Table S3. Predictors of urinary markers in sanctuary and wild chimpanzees.** Markers are corrected for specific gravity and log transformed. Results are from models that exclude the *age***facility* interaction, since this was never a significant predictor.

|  | **uNeopterin** | **usuPAR** | **uIsoprostanes** | **uOHdG** |
| --- | --- | --- | --- | --- |
| Time of day  *(scaled)* | *Est*. = -0.05  *SE =* 0.01  *t =* -3.78  *p* < 0.001 | *Est*. = -0.13  *SE =* 0.06  *t =* -2.24  *p* = 0.03 | *Est*. = -0.04  *SE =* 0.02  *t =* -2.25  *p* = 0.02 | *Est*. = 0.07  *SE =* 0.02  *t =* 4.30  *p* < 0.001 |
| Sex  *(reference = female)* | *Est*. = 0.02  *SE =* 0.04  *t =* 0.60  *p* = 0.55 | *Est*. = 0.33  *SE =* 0.12  *t =* 2.77  *p* = 0.01 | *Est*. = 0.16  *SE =* 0.04  *t =* 3.94  *p* < 0.001 | *Est*. = -0.24  *SE =* 0.03  *t =* -6.96  *p* < 0.001 |
| Age | *Est*. < 0.01  *SE* < 0.01  *t =* 0.52  *p* = 0.61 | *Est*. = 0.01  *SE =* 0.01  *t =* 2.10  *p* = 0.04 | *Est*. > -0.01  *SE <* 0.01  *t =* -0.44  *p* = 0.66 | *Est*. > -0.01  *SE* < 0*.*01  *t =* -0.59  *p* = 0.56 |
| Facility  *(reference = wild)* | *Est*. = 1.00  *SE* = 0.12  *t =* 7.88  *p* < 0.001 | *Est*. = 0.32  *SE =* 0.17  *t =* 1.92  *p* = 0.06 | *Est*. = 0.35  *SE* = 0.09  *t =* 4.07  *p* = 0.001 | *Est*. > -0.01  *SE* = 0.10  *t =* -0.07  *p* = 0.95 |

**Table S4. Predictors of urinary and serum markers in sanctuary chimpanzees.** Urinary markers are corrected for specific gravity, and all markers are log transformed. Results are from full models.

|  | **uNeopterin** | **usuPAR** | **uIsoprostanes** | **uOHdG** | **sCRP** | **sIL6** |
| --- | --- | --- | --- | --- | --- | --- |
| Time of day  *(scaled)* | *Est*. = 0.03  *SE =* 0.05  *t =* 0.53  *p* = 0.60 | *Est*. = 0.22  *SE =* 0.18  *t =* 1.1  *p* = 0.23 | *Est*. = -0.03  *SE =* 0.04  *t =* -0.79  *p* = 0.43 | *Est*. = 0.01  *SE =* 0.07  *t =* 0.08  *p* = 0.93 | *NA* | *NA* |
| Sex  *(reference = female)* | *Est*. = -0.05  *SE =* 0.09  *t =* -0.50  *p* = 0.62 | *Est*. = 0.44  *SE =* 0.30  *t =* 1.48  *p* = 0.15 | *Est*. = 0.03  *SE =* 0.07  *t =* 0.44  *p* = 0.66 | *Est*. = -0.21  *SE =* 0.14  *t =* -1.49  *p* = 0.14 | *Est*. = -0.33  *SE =* 0.23  *t =* -1.40  *p* = 0.17 | *Est*. = 0.57  *SE =* 0.17  *t =* 3.36  *p* < 0.01 |
| Age | *Est*. = 0.01  *SE =* 0.01  *t =* 1.02  *p* = 0.31 | *Est*. = -0.01  *SE =* 0.02  *t = -*0.34  *p* = 0.74 | *Est*. < 0.01  *SE =* 0.01  *t =* 0.19  *p* = 0.85 | *Est*. = -0.01  *SE =* 0.01  *t =* -0.61  *p* = 0.55 | *Est*. = -0.02  *SE =* 0.02  *t =* -0.87  *p* = 0.39 | *Est*. = 0.02  *SE =* 0.01  *t =* 1.24  *p* = 0.22 |
| Site  *(reference = Ngamba)* | *Est*. = 0.24  *SE* = 0.66  *t =* 0.36  *p* = 0.75 | *NA* | *Est*. = 0.10  *SE* = 0.22  *t =* 0.45  *p* = 0.69 | *Est*. = -0.26  *SE* = 0.39  *t =* -0.66  *p* = 0.56 | *Est*. = 1.41  *SE* = 1.51  *t =* 0.93  *p* = 0.52 | *Est*. = 0.75  *SE* = 0.21  *t =* 3.49  *p* = 0.001 |

**Table S5. Predictors of urinary markers in wild chimpanzees.** All markers are corrected for specific gravity and log transformed. Results are from full models.

|  | **uNeopterin** | **usuPAR** | **uIsoprostanes** | **uOHdG** |
| --- | --- | --- | --- | --- |
| Time of day  *(scaled)* | *Est*. = -0.06  *SE =* 0.01  *t =* -4.22  *p* = < 0.001 | *Est*. = -0.13  *SE =* 0.06  *t =* -2.24  *p* = 0.03 | *Est*. = -0.04  *SE =* 0.02  *t =* -2.19  *p* = 0.03 | *Est*. = 0.08  *SE =* 0.02  *t =* 4.74  *p* < 0.001 |
| Sex  *(reference = female)* | *Est*. = 0.05  *SE =* 0.04  *t =* 1.08  *p* = 0.29 | *Est*. = 0.31  *SE =* 0.13  *t =* 2.39  *p* = 0.02 | *Est*. = 0.19  *SE =* 0.05  *t =* 3.90  *p* < 0.001 | *Est*. = -0.24  *SE =* 0.04  *t =* -6.79  *p* <0.001 |
| Age | *Est*. < 0.01  *SE* < 0.01  *t =* 0.28  *p* = 0.78 | *Est*. = 0.02  *SE* = 0.01  *t =* 2.39  *p* = 0.02 | *Est*. = -0.001  *SE <* 0.01  *t = -*0.49  *p* = 0.63 | *Est*. > -0.01  *SE* < 0.01  *t =* -0.58  *p* = 0.56 |

**
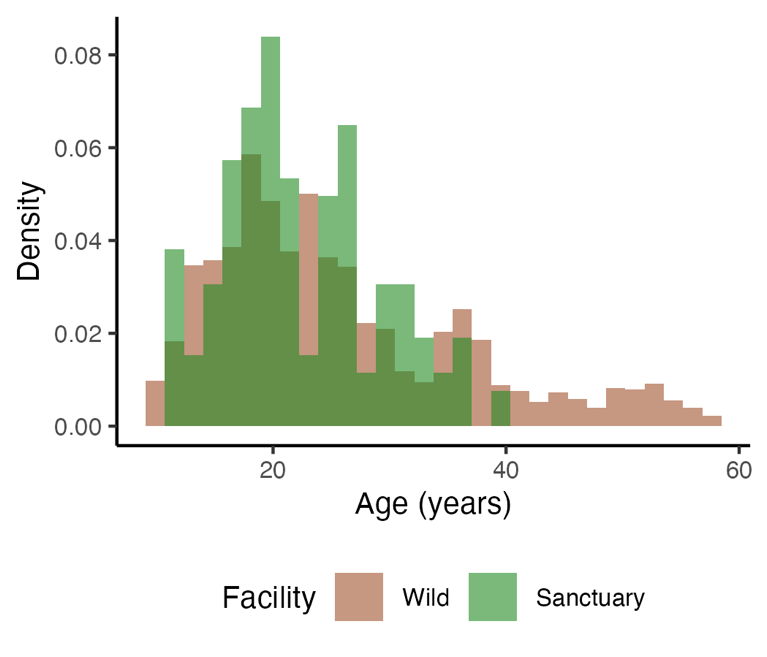
**

**Figure S1. Age distributions of sanctuary versus wild chimpanzees.**

**
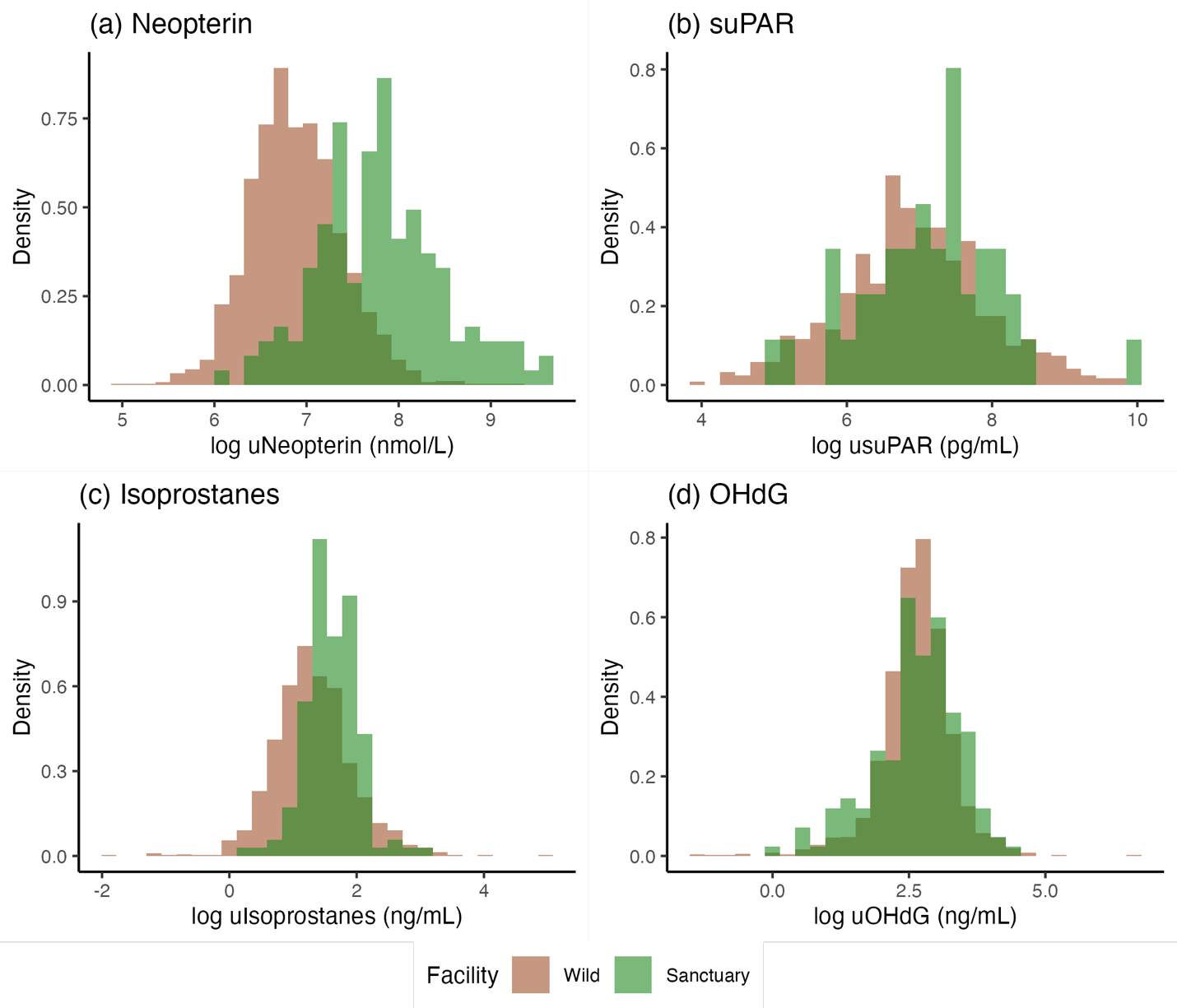
**

**Figure S2. Histograms of urinary markers in sanctuary versus wild chimpanzees.** All markers are corrected for specific gravity and log transformed.

**
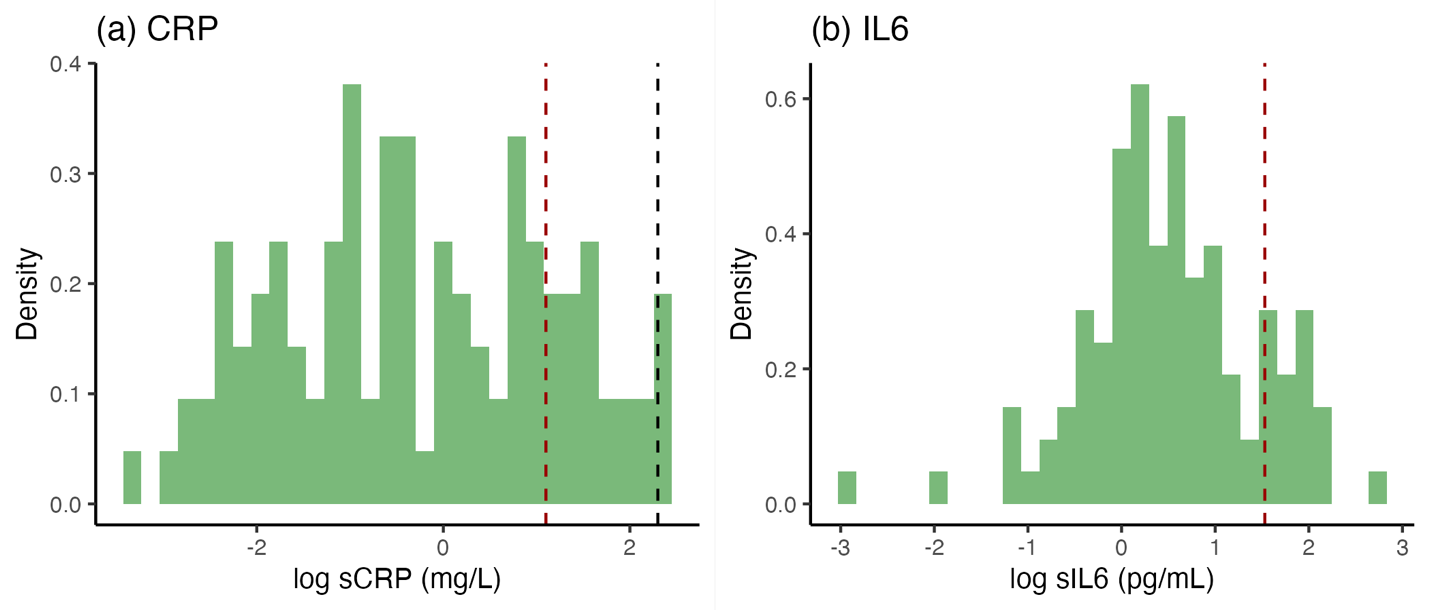
**

**Figure S3. Histograms of serum markers in sanctuary chimpanzees.** All markers are log transformed. Red dashed lines indicate human cutoffs for clinical elevation (over 3 mg/L for CRP and over 4.64 pg/mL for IL6). Black dashed line indicates human cutoff for acute infection (over 10 mg/L for CRP). Risk thresholds are from (Pearson et al., 2003).
